# Transcriptome Analysis Reveals the Role of Plant Hormone Signal Transduction Pathways in the Drought Stress Response of *Hemerocallis middendorffii*

**DOI:** 10.3390/plants14071082

**Published:** 2025-04-01

**Authors:** Ying Qian, Haihang Yu, Siyu Lu, Yun Bai, Yuan Meng, Lifei Chen, Lin Wu, Yunwei Zhou

**Affiliations:** College of Horticulture, Jilin Agricultural University, Changchun 130118, China; qiany9071@163.com (Y.Q.); yuhaihang1117@163.com (H.Y.); l1964561250@163.com (S.L.); yunb@jlau.edu.cn (Y.B.); lfchen@jlau.edu.cn (L.C.)

**Keywords:** *Hemerocallis middendorffii*, drought stress, transcriptome, transcription factors, plant hormone signal transduction

## Abstract

Drought stress is a significant environmental factor that can impede plant growth and ornamental quality. *Hemerocallis middendorffii*, a drought-tolerant garden plant, has attracted attention for its ornamental value and application prospects. To investigate the molecular mechanism of drought stress resistance of *H*. *middendorffii*, this study employed 20% polyethylene glycol (PEG) 6000 to simulate drought stress. Leaves and roots of *H*. *middendorfii* were subjected to 24 h treatment and followed by transcriptome sequencing. Analysis revealed 8796 and 3401 differentially expressed genes (DEGs) in leaves and roots. The major biological processes and key molecular pathways activated under drought stress in *H*. *middendorffii* were revealed by Gene Ontology (GO) and Kyoto Encyclopedia of Genes and Genomes (KEGG) enrichment analyses. The focus of this analysis was on the gene expression changes within plant hormone signal transduction pathway. Additionally, drought-associated transcription factor families such as AP2/ERF, WRKY, MYB, bHLH, NAC, and bZIP were identified among DEGs. Furthermore, potential regulatory relationships of the above transcription factors (TFs) with functional genes in the abscisic acid (ABA) and jasmonic acid (JA) signalling pathways were analysed using correlation network prediction. This research establishes the groundwork for subsequent exploration of drought-responsive gene expression and regulatory patterns in *H*. *middendorfii* and provides an importance for the systematic study of its drought-resistant molecular mechanism.

## 1. Introduction

*Hemerocallis middendorffii* is a perennial herbaceous ornamental plant that belongs to the genus *Hemerocallis* in the family Asphodelaceae, and it serves as an important hybrid parent for the breeding of modern daylily cultivars. The plant material is widely distributed in the three northeastern provinces and has abundant wild plant resources. It features excellent qualities such as strong drought resistance, vivid flower colours, an early flowering period, and tolerance to rough management. *H*. *middendorffii* is extensively utilized in flower beds, flower borders, and woodland edges. It is regarded as an ideal choice for urban landscaping and beautiful countryside construction [1]. At this stage, research on *Hemerocallis* plant mainly focuses on genetic diversity [2], floral organ development [3], aromatic compounds [4], and physiological mechanisms of stress tolerance [1,5,6]. However, there exists a paucity of research at the transcriptome level concerning the molecular mechanisms involved in drought stress in the *H*. *middendorffii*.

Drought is now widely acknowledged to be among the primary abiotic stresses exerting an influence on plant growth and ornamental traits. This results from several elements, encompassing the gradual increase in global temperature, the increasing scarcity of freshwater resources, and the multiple challenges posed by human activities [7]. The growth and development of plants is restricted by drought, which has serious consequences for the stability of the ecosystem and the sustainability of agricultural production. To cope with and withstand changes in extreme environments, plants have evolved sophisticated mechanisms for sensing unfavourable conditions and are capable of responding promptly for regulating their growth and development [8,9]. Plants have evolved a range of mechanisms to cope with the adverse effects of drought stress, including changes in their own morphological structure, physiological and biochemical aspects, drought-resistant gene expression, hormone synthesis, and osmotic regulatory substances. The plant exhibits several adaptive features, including a reduction in growth rate, partial stomatal closure to minimise the loss of water, and the accumulation of free radicals [10,11,12,13].

The recurrent occurrence of drought has profoundly impacted the global ecological balance, necessitating the investigation of drought-resistant adaptive mechanisms and the cultivation of novel drought-tolerant plant varieties. This has become a demanding and urgent scientific research endeavour. The leaves of the above-ground part of the plant are the first to sense the loss of water, while the roots of the underground part are the first organs affected by drought [14]. Consequently, research concerning plant drought resistance has primarily concentrated on the analysis of plant organs, specifically leaves and roots [15]. Abscisic acid (ABA) is a pivotal phytohormone for plant growth, development, and the regulation of abiotic stresses [16]. The stimulation of ABA production and accumulation in plant organs is induced by drought stress, with the resultant activation of downstream signalling pathways [17]. Furthermore, a number of other phytohormones have also been recognized as having a fundamental influence on drought stress response. These include brassinosteroids (BR), cytokinin (CTK), and auxin (IAA), which have been found to crosstalk with ABA in order to regulate that response [18,19]. Transcription factors (TFs) serve a significant function in signal transduction pathways, interacting with the promoter regions of target genes to modulate their expression. The families of TFs most implicated in the realm of plant drought stress are chiefly bZIP, AP2/ERF, MYB, and WRKY, amongst others [20,21]. The aforementioned TFs have been demonstrated to regulate the expression of genes associated with the response to drought stress, thereby improving drought tolerance in plants. TFs respond to different adversity responses by regulating related genes in different hormone signalling pathways. The present study set out the finding that *OsbZIP62* improves drought tolerance in rice by participating in the ABA signalling pathway and interacting with protein kinases (SAPKs) to regulate the expression of related genes [22]. *GbWRKY1* depends on the interaction of JAZ1 and ABI1 to negatively regulate ABA signalling and inhibit salt and drought tolerance in plants [23].

Transcriptome sequencing technology has become a significant instrument for elucidating the molecular mechanisms of abiotic stresses. It has been extensively utilised in research endeavours pertaining to drought stress signalling pathways and the prediction of gene function in plants [24]. At the present time, the molecular mechanisms underlying drought stress responses have been analysed using transcriptome sequencing in many plant species, including Lanzhou lily [25], rose [26], and iris [27]. Certain advancements have occurred in the investigation of molecular mechanisms underlying plant response to drought stress, but the change rule of genes and response mechanisms of *H*. *middendorffii* under drought stress are still unknown. The objective of this study was to ascertain the alterations in the expression profiles of differentially expressed genes (DEGs) in leaves and roots of *H*. *middendorffii* by simulating drought stress with polyethylene glycol (PEG) 6000 and to reveal the key metabolic pathways in response to drought stress. A particular focus was on exploring the potential regulatory relationship between drought-resistant TFs and the functional genes within ABA and jasmonic acid (JA) signalling pathways. The findings of this research will offer a conceptual foundation for the comprehensive understanding of the molecular mechanism of drought tolerance in leaves and roots of *H*. *middendorffii*. Simultaneously, the study will establish a framework for the excavation of drought-tolerant key genes and the further elucidation of molecular regulatory mechanisms.

## 2. Results

### 2.1. Transcriptome Sequencing Data and Assembly Statistics

In the present study, the gene expression patterns of *H*. *middendorffii* under drought stress conditions were analysed using transcriptome sequencing technology. The 12 cDNA libraries were constructed from six samples of leaf tissue (PL: leaf tissue under drought stress, CL: control leaf tissue) and six samples of root tissue (PR: root tissue under drought stress, CR: control root tissue). A total of 277,689,578 raw reads were obtained. The subsequent removal of low-quality data from the original sequence of data resulted in the attainment of 271,604,426 clean reads. The range of the GC content of the samples was from 44.05% to 47.03%, with an error rate of only 0.03%; Q20 was higher than 97.43% and Q30 was higher than 92.73% (Table S1). Trinity assembled a total of 194,376 unigenes, with an average length of 829 bp and an N50 of 1057 bp (Table S2). In conclusion, the data derived from the sequencing of transcriptomes were of a high quality and could be utilised for further subsequent analyses.

### 2.2. Analysis of DEGs

**Figure 1:**
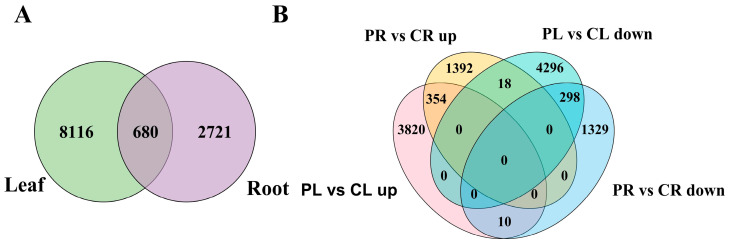
Quantity and distribution of DEGs in leaves and roots of *H*. *middendorffii* under drought stress. (**A**) Venn diagram analysis of DEGs; (**B**) Venn diagram analysis of upregulated and downregulated DEGs.

In order to investigate alterations in genetic expression profiles in leaves and roots of the *H*. *middendorffii* under drought stress, DEGs were screened and analysed. The results demonstrated that 8796 and 3401 DEGs were obtained in leaves and roots, of which 680 genes were differentially expressed in both leaf and root tissues. Furthermore, there were 8116 and 2721 DEGs with specificity in leaves and roots (Figure 1A). Among these genes which demonstrated differential expression, 4184 genes were found to be upregulated, whilst 4612 genes were downregulated in the leaves. For the roots, 1764 genes were upregulated, and 1637 genes were downregulated (Figure 1B). The findings suggest that the leaves and roots of *H*. *middendorffii* may adopt different response mechanisms and strategies in resisting drought stress.

### 2.3. Gene Ontology (GO) Enrichment Analysis of DEGs

**Figure 2:**
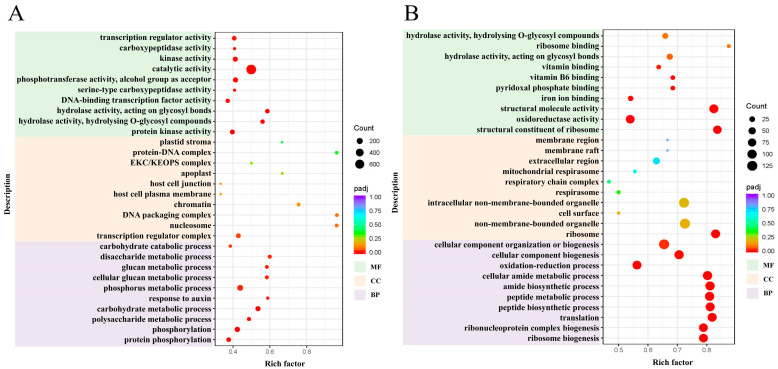
GO enrichment analysis of DEGs under drought stress; (**A**) leaves; (**B**) roots.

To enhance comprehension of the prospective biological functions of DEGs in *H*. *middendorffii* in the context of drought stress, GO functional annotation and enrichment analyses were conducted on DEGs in leaf and root tissues. The objective of this analysis was to reveal significant variations in biological processes, cellular components, and molecular functions. A statistical analysis was conducted on the top 30 GO terms, which were ranked for both leaf and root tissues. The screening criterion for significant enrichment was *p*-value ≤ 0.05. GO enrichment analyses demonstrated that 4494 DEGs in leaves and 2450 DEGs in roots were annotated to significantly enriched GO terms. In leaves, drought-responsive genes were significantly enriched mainly for protein phosphorylation, phosphorylation, polysaccharide metabolic process, and carbohydrate metabolic process in biological processes, followed by protein kinase activity, hydrolase activity, hydrolysing O-glycosyl compounds, hydrolase activity, acting on glycosyl bonds in molecular functions and mainly transcription regulator complex, and so on, in cellular components (Figure 2A). In roots, drought-responsive genes were significantly enriched mainly in ribosome biogenesis and ribonucleoprotein complex biogenesis of biological processes, structural constituent of the ribosome, oxidoreductase activity, and structural molecule activity of molecular functions and the ribosome, and so on, in cellular components (Figure 2B). In summary, the drought stress response mechanism of *H*. *middendorffii* is primarily focused on signal transduction, energy metabolism, and other biological functions.

### 2.4. Kyoto Encyclopedia of Genes and Genomes (KEGG) Enrichment Analysis of DEGs

**Figure 3:**
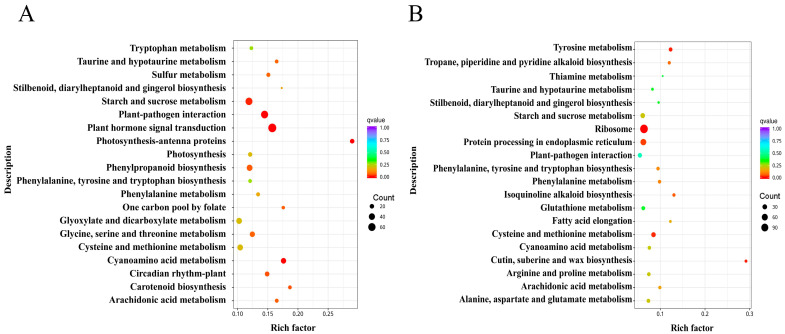
KEGG enrichment analysis of DEGs under drought stress; (**A**) leaves; (**B**) roots.

This research annotated and analysed KEGG metabolic pathways of DEGs in response to drought stress in *H*. *middendorffii*. KEGG pathway enrichment analysis demonstrated that 1529 DEGs were annotated to 117 KEGG pathways in leaves, which exhibited significant enrichment in pathways such as plant hormone signal transduction, photosynthesis–antenna proteins, plant–pathogen interaction, cyanoamino acid metabolism, and starch and sucrose metabolism (Figure 3A). In roots, a sum of 865 DEGs were annotated to 100 KEGG pathways, with the main significantly enriched pathways being ribosome, cutin, suberine and wax biosynthesis, tyrosine metabolism, cysteine, and methionine metabolism and protein processing in endoplasmic reticulum (Figure 3B). The findings suggest that leaves and roots may employ divergent mechanisms to withstand drought stress, with the significantly enriched metabolic pathways displaying a strong correlation with the response to drought stress in *H*. *middendorffii*.

### 2.5. Identification and Analyzation of Differentially Expressed TFs

**Figure 4:**
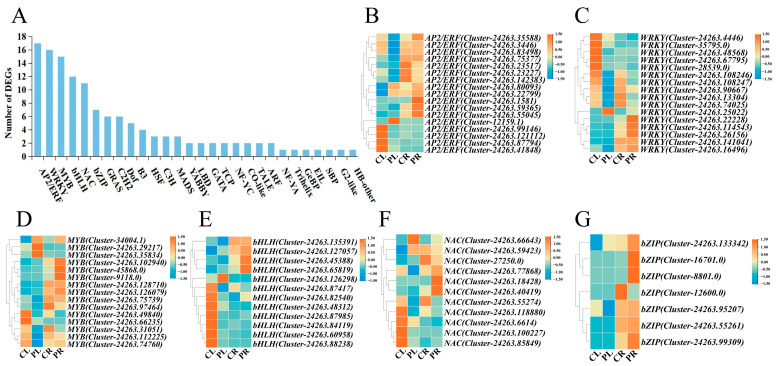
Distribution and expression patterns of TFs in response to drought stress; (**A**) classification of identified transcription factor families; (**B**) heatmap of *AP2/ERF* expression; (**C**) heatmap of *WRKY* expression; (**D**) heatmap of *MYB* expression; (**E**) heatmap of *bHLH* expression; (**F**) heatmap of *NAC* expression; (**G**) heatmap of *bZIP* expression.

TFs represent pivotal regulatory elements that modulate the expression of genes responsive to drought stress, playing a critical role in plant responses to this environmental stressor [28]. Overall, 131 TFs belonging to 28 distinct families of TFs were identified through analysis of the PlantTFDB database and transcriptome sequence alignment. Among the identified transcription factor families, AP2/ERF was the most abundant with 17 members, followed by WRKY (16), MYB (15), bHLH (12), NAC (11), bZIP (7), GRAS (6), C2H2 (6), and Dof (5) (Figure 4A). We analysed the expression patterns in the form of heatmaps for the most numerous AP2/ERF, WRKY, MYB, bHLH, NAC, and bZIP families. The AP2/ERF family showed three DEGs upregulated and 12 DEGs downregulated for expression in leaves, and all three DEGs were upregulated for expression in roots (Figure 4B). The WRKY family exhibited a single DEG that was upregulated, with the remaining 11 being downregulated for expression in leaves. Moreover, four DEGs showed upregulated expression, while one exhibited downregulated expression in roots (Figure 4C). In the MYB family, there were six DEGs upregulated and six DEGs downregulated in leaves, while in roots, three DEGs were upregulated, and two DEGs were downregulated (Figure 4D). The bHLH and NAC families exhibited two and four DEGs being upregulated, and seven and five DEGs being downregulated, for expression in leaves, respectively. Moreover, three DEGs were upregulated for expression in both roots (Figure 4E,F). The bZIP family was observed to comprise a DEG that was upregulated and two DEGs that were downregulated for expression in leaves. In addition, three DEGs were upregulated and one DEG was downregulated for expression in roots (Figure 4G). In summary, it can be seen that the majority of the DEGs of the AP2/ERF, WRKY, MYB, bHLH, NAC, and bZIP families exhibited a decreasing trend of expression in leaves and an increasing trend of expression in roots.

### 2.6. Analysis of the Expression Pattern of Relevant DEGs on the Plant Hormone Signal Transduction Pathway

**Figure 5:**
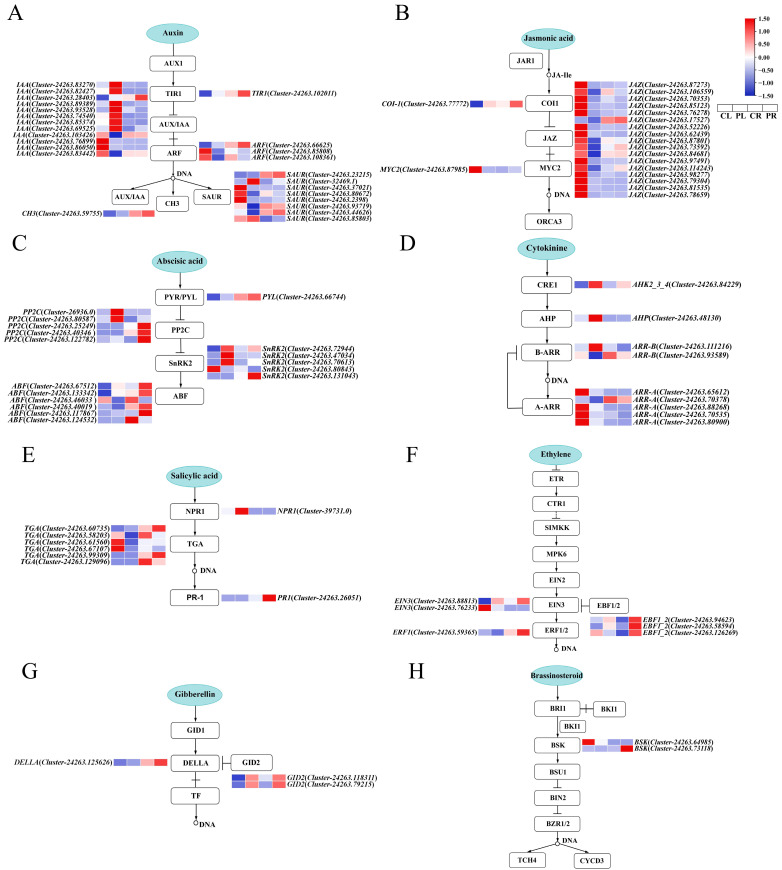
Expression pattern of DEGs associated with the plant hormone signal transduction pathway under drought stress; (**A**) the IAA signalling pathways; (**B**) the JA signalling pathways; (**C**) the ABA signalling pathways; (**D**) the CTK signalling pathways; (**E**) the SA signalling pathways; (**F**) the ET signalling pathways; (**G**) the GA signalling pathways; (**H**) the BR signalling pathways.

An analysis of the expression patterns of DEGs associated with the plant hormone signal transduction pathway of the *H*. *middendorffii* revealed the identification of 74 and 22 DEGs in the leaves and roots. These genes were found to be enriched in eight distinct hormonal signalling pathways, including IAA, gibberellin (GA), ABA, and so on. The greatest count of DEGs were involved in the IAA, ABA, and JA signalling pathways. Overall, 25 DEGs were enriched in the IAA signalling pathway, with most of the DEGs clustered in two genes, *IAA* and *SAUR*, and of the 12 *IAA* genes, only one *IAA* gene (*Cluster-24263.69525*) was significantly upregulated in both leaf and root tissues, while the remaining genes were upregulated in seven and downregulated in four cases in leaves. The *SAUR* gene exhibited one upregulated expression in roots, while leaves exhibited two upregulated and five downregulated expressions (Figure 5A). All 19 DEGs involved in the JA signalling pathway presented differential expression patterns in leaves, including an upregulated *COI-1* gene, 17 downregulated *JAZ* genes, and one downregulated *MYC2* gene (Figure 5B). The present study identified a total of 17 DEGs involved in the ABA signalling pathway, including a *PYL* gene that was upregulated in leaves and two and four *PP2C* genes that were upregulated in both leaves and roots. Among the five *SnRK2* genes, only one *SnRK2* (*Cluster-24263.80843*) was found to be downregulated in terms of expression in both roots and leaves. *ABF* genes exhibited divergent patterns of expression, with two upregulated and two downregulated expressions in both leaves and roots. Specifically, *Cluster-24263.67512* and *Cluster-24263.46033* were identified as being upregulated and downregulated in both tissues, respectively (Figure 5C).

In addition, the response of *H*. *middendorffii* to drought stress involved the CTK, salicylic acid (SA), ethylene (ET), GA, and BR signalling pathways, with nine, eight, six, and two DEGs. Within the CTK signalling pathway, an *ARR-B* gene (*Cluster-24263.111216*) was found to be upregulated expression in leaves and downregulated expression in roots, and five *ARR-A* genes had downregulated expression in leaves (Figure 5D). In the SA signalling pathway, the highest number of DEGs enriched *TGA* genes was found, with one showing upregulated and three showing downregulated expression in leaves, and two showing upregulated and one showing downregulated expression in roots (Figure 5E). In the ET signalling pathway, three *EBF1_2* genes showed upregulated expression in roots, and an *ERF1* gene demonstrated downregulated expression in leaves but upregulated expression in roots (Figure 5F). Within the GA signalling pathway, it was observed that a *GID2* gene was upregulated in leaves, and a *GID2* gene was upregulated in roots, while a *DELLA* gene was specifically upregulated in leaves (Figure 5G). In the BR signalling pathway, two *BSK* genes were identified, one of which was downregulated for expression in leaves, and the other upregulated for expression in roots (Figure 5H).

### 2.7. Correlational Network Analysis of Functional Genes Associated with ABA and JA Signal Transduction Pathways in Correlation with TFs

**Figure 6:**
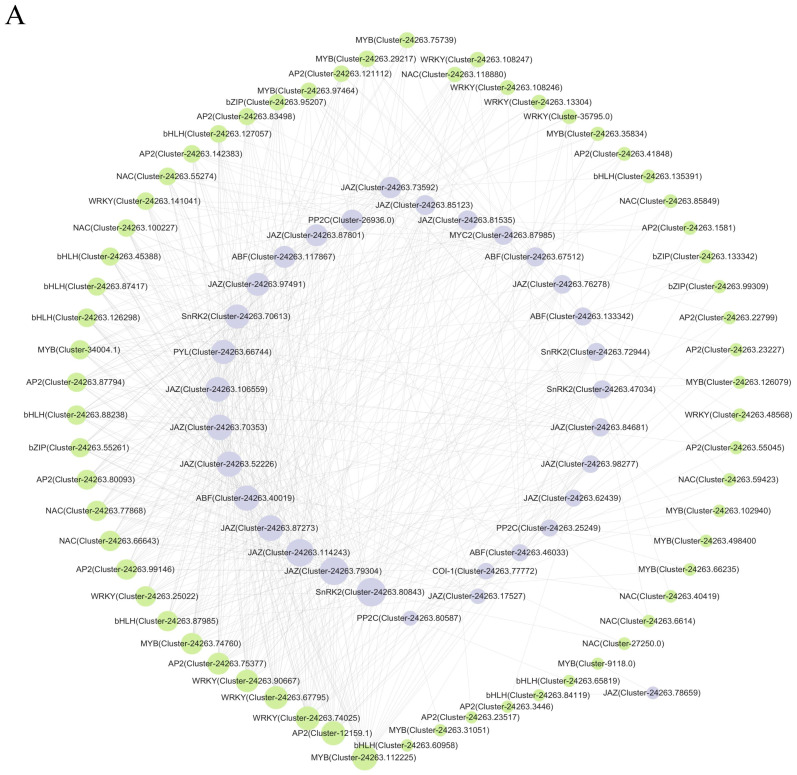

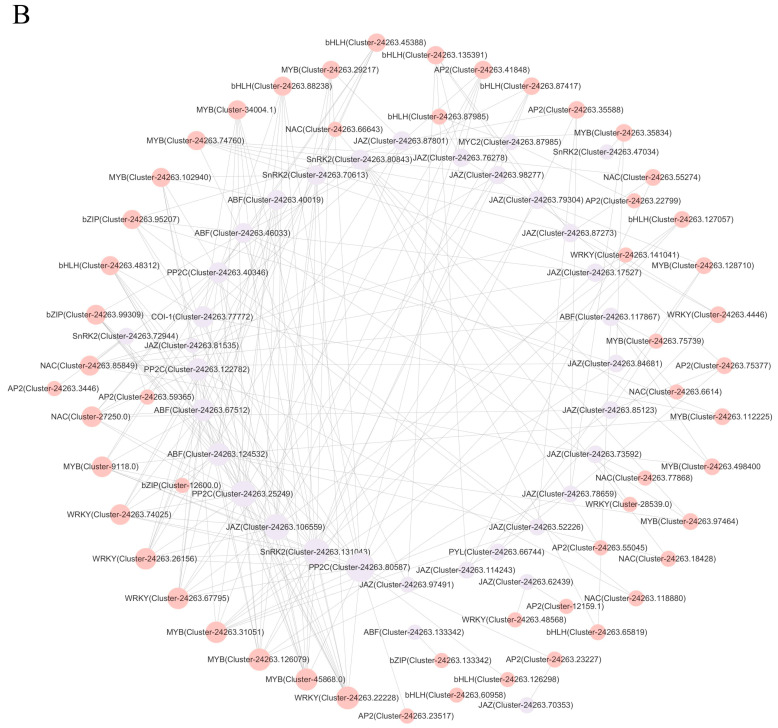
Network analysis of correlations between AP2/ERF, WRKY, MYB, bHLH, NAC, bZIP, and functional genes connected to the ABA and JA signal transduction pathways. (**A**) leaves; (**B**) roots.

As demonstrated by related studies, the signalling pathways of ABA and JA have been shown to induce stomatal closure and improve drought tolerance in plants [29,30]. The present study employed AP2/ERF, WRKY, MYB, bHLH, NAC, and bZIP transcription factors for the purpose of correlation network analysis with functional genes on the ABA and JA pathways. The prediction results showed that a total of 62 TFs had possible regulatory relationships with 32 functional genes in leaves (Figure 6A). Major functional genes regulated by AP2/ERF, WRKY, MYB, bHLH, NAC, and bZIP included *SnRK2* (*Cluster-24263.80843*), *JAZ* (*Cluster-24263.79304*), *JAZ* (*Cluster-24263.114243*), *JAZ* (*Cluster-24263.87273*), *JAZ* (*Cluster-24263.52226*), *ABF* (*Cluster-24263.40019*), and others. A total of 54 TFs in the root had probable regulatory relationships with 35 DEGs (Figure 6B). It has been determined that the major functional genes regulated by AP2/ERF, WRKY, MYB, bHLH, NAC, and bZIP in roots include *PP2C* (*Cluster-24263.80587*), *SnRK2* (*Cluster-24263.131043*), *PP2C* (*Cluster-24263.25249*), *JAZ* (*Cluster-24263.106559*), and others.

### 2.8. Validation of RNA-Seq Data by qRT-PCR of DEGs

**Figure 7:**
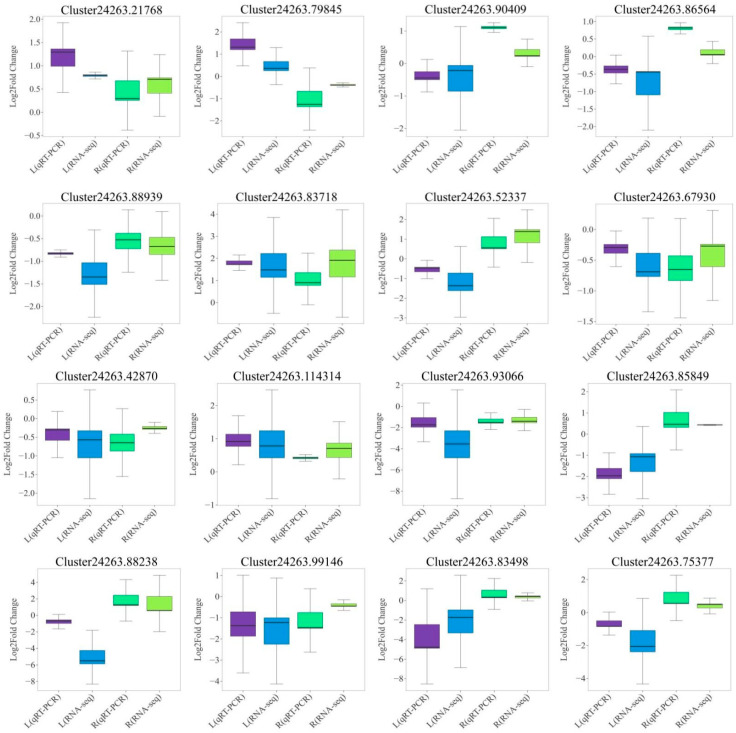
Expression patterns of 16 DEGs screened by qRT-PCR validation.

To ensure the authenticity of the leaves and roots transcriptome data of *H*. *middendorffii* under 20% PEG-6000 simulated drought stress, qRT-PCR validation analyses of 16 randomly selected DEGs associated with drought stress were performed. The findings indicated that the selected DEGs showed similar gene expression trends in the qRT-PCR results and transcriptome sequencing results (Figure 7). The aforementioned results suggest that the RNA-Seq data are of a higher quality and thus suitable for subsequent analyses.

## 3. Discussion

Drought is widely regarded as a major stress factor, impacting plant growth, development, and appearance quality [31]. With the accelerated development of the global economy and the continuous expansion of urban areas, the water consumption and cost of landscape management are increasing year by year. Consequently, the selection of plants with superior ornamental characteristics, along with those exhibiting water-saving and drought-resistant qualities, is poised to become the prevailing trend in future garden construction. *H*. *middendorffii* is a drought-tolerant ornamental plant, and it is of great significance to reveal its drought-resistant molecular mechanism and add new plant resources for the construction of water-saving gardens.

Transcriptome sequencing provides comprehensive and rapid access to all transcript information and has become an effective tool for analysing plant response mechanisms to abiotic stresses [32]. In instances where genomic information is lacking, the employment of transcriptome sequencing technology enables the acquisition of a substantial volume of transcript sequence data. And based on this, functional gene mining of plants was undertaken in order to disclose the underlying mechanisms of desirable plant traits [33]. The present study analysed the DEGs of *H*. *middendorffii* under drought stress. Significant changes in DEGs were observed in both leaves and roots. This phenomenon may be attributable to a series of stress responses exhibited by plants, which are initiated in order to mitigate water loss in response to drought stress. The number of DEGs was found to be greater in leaves than in roots, suggesting that there may be differences in the mechanism of response to drought stress in these two organs. Concurrently, analysis revealed 680 DEGs shared between the two tissues, thereby further suggesting that these DEGs may serve a pivotal role in the response of leaves and roots to drought stress.

GO and KEGG pathway enrichment analyses of the DEGs in response to drought stress in *H*. *middendorffii* were performed to identify key pathways associated with this response. In this current study, the pathways that were identified as highly enriched with DEGs in leaves primarily included plant hormone signal transduction, photosynthesis-antenna proteins, cyanoamino acid metabolism, and starch and sucrose metabolism. This finding suggests that signal transduction, energy supply, and depletion may be the primary strategies for coping with drought stress in *H*. *middendorffii*. The plant hormone signal transduction pathway was identified as the most prominently enriched pathway, and it is hypothesised that this pathway is the most important one for drought tolerance in the *H*. *middendorffii*. In root tissues, the pathway with the highest enrichment of DEGs is the ribosome, which contains the highest number of DEGs. The process of translating mRNA into protein by ribosomes represents a pivotal step in gene expression [34]. As demonstrated in previous studies, there is a differential expression of ribosomal protein genes in plants in response to drought stress, with enhanced resistance being observed in transgenic plants [35]. In this particular study, it was found that the number of DEGs enriched in this pathway in roots was significantly higher than in leaves. This phenomenon may be attributable to the capacity of the *H*. *middendorffii* roots to accumulate greater quantities of nutrients, thereby supplying energy to the ribosomal pathway.

The plant hormone signal transduction pathway exerts a primary regulatory influence over plant physiology by means of a complex network of hormone signals, including ABA, IAA, JA, and CTK, thereby enabling plants to cope with drought stress [36]. ABA-mediated signalling pathways have been demonstrated to be of significance in plants drought stress response, exerting an important influence on stomatal closure and the expression of resistance genes in plants [37]. Within this pathway, the upstream *PYR/PYL* (ABA receptor) interaction with *PP2C* results in the inhibition of the negative regulator *PP2C*, thus reducing its inhibitory effect on *SnRK2* [38]. It has previously been demonstrated that the over-expression of the *BdSnRK2.9* gene leads to an enhancement in drought tolerance in transgenic plants [39]. In the current study, five *PP2C* genes were identified in both leaves and roots. All of these were found to be significantly upregulated, and most of the *SnRK2* genes were similarly upregulated. This is probably due to the fact that *SnRK2* genes are regulated by TFs in response to drought stress. IAA signal transduction primarily regulates processes such as plant root growth, leaf development, and lightward growth [40]. In this paper, five *SAUR* and four *IAA* genes were downregulated for expression in leaves, likely due to the inhibition of growth hormone synthesis, which resulted in slowing down of biomass accumulation, thus improving the drought tolerance of the plants. It has been established that the JA signalling pathway is intimately associated with the mitigation of drought stress. The JAZ protein family is a negative regulator in this pathway [41]. Earlier research has established that the overexpression of *OsJAZ1* results in a reduction in drought tolerance in rice through the regulation of the ABA and JA signalling pathways [42]. In parallel, the *MYC2* has been shown to significantly reduce the response to dehydration stress [43]. We found that 17 *JAZ* and one *MYC2* were downregulated expression under drought stress in *H*. *middendorffii* leaves. It is hypothesised that the *JAZ* gene may have a significant role in the response of *H*. *middendorffii* to drought stress.

As evidenced by numerous studies, the TF families AP2/ERF, bZIP, MYB, NAC, WRKY, and bHLH have been identified as being pivotal regulators in the context of drought stress responses [44,45,46]. In our investigation, 131 DEGs encoding TFs were identified in leaves and roots. These DEGs were predominantly distributed among the AP2/ERF, WRKY, MYB, bHLH, NAC, and bZIP families. The results indicate that the expression of numerous TF families is enhanced by drought stress, and the expression of the common TF families plays a significant function in the response of *H*. *middendorffii* to drought stress. A related study reported that the *PtoERF15* directly regulated the expression of *PtoMYC2* to modulate the JA pathway and enhance drought tolerance in *Populus* [47]. *GmWRKY54* directly bonded to the promoters of *PYL8*, *SRK2A*, *CIPK11*, and *CPK3* and activated their expression, which improved drought tolerance in soybean [48]. The *MdMYB44-like* has been shown to repress *MdPP2CA* transcription and enhance ABA mediated drought and salt tolerance [49]. *OsbHLH148* enhanced drought tolerance in rice by interacting with *OsJAZ* protein [50]. *RtNAC055* was subjected to direct regulation by *RtMYC2*, with the result that it promoted plant drought tolerance by regulating stomatal closure through JA/hydrogen peroxide signalling [51]. *OsbZIP23* positively regulated *OsPP2C49* and regulated ABA signalling [52]. Through the correlation network prediction, it was found that AP2/ERF, WRKY, MYB, bHLH, NAC, and bZIP may influence the plant’s drought tolerance by regulating the expression of downstream genes such as *PP2C*, *JAZ* and *SnRK2*. However, further investigation is required to elucidate the intricate regulatory relationships among these factors.

## 4. Materials and Methods

### 4.1. Material and Drought Stress Treatment

The experimental material employed in this study was the *H*. *middendorffii*, which was supplied by the Ornamental Plant Resource Nursery of Jilin Agricultural University. In the greenhouse, single plants displaying robust and uniform growth were selected for planting in 10 cm × 13 cm pots. The cultivation substrate was composed of a blend of peat, garden soil, and perlite, with a proportion of 3:1:1. Subsequent to the cessation of the establishment period, the plants were arbitrarily allocated to two groups. Equal quantities (250 mL) of piped water and 20% PEG-6000 were administered to the roots of the plants, as determined by the preliminary experiment, with piped water serving as the control treatment. A total of 60 plants were utilised in the experimental setup, with 10 pots per treatment configured with three biological replicates. After the completion of a 24 h period of treatment, the leaves and roots were collected separately. They were then rapidly snap-frozen using liquid nitrogen, after which they were stored in a −80 °C refrigerator for subsequent use.

### 4.2. Total RNA Extraction, cDNA Library Construction, Sequencing and De Novo Assembly from the Hemerocallis middendorffii

The extraction of total RNA from leaves and roots of *H*. *middendorffii* was conducted in accordance with the TransZol Up Plus RNA Kit (TransGen Biotech, Wuhan, China) as outlined by the manufacturer. Subsequently, an Agilent 2100 bioanalyzer (Agilent, Santa Clara, CA, USA) was employed to assess the integrity of the extracted RNA. After passing the test, double-stranded cDNA was synthesised by reverse transcription, and suitable fragment sizes were screened for PCR amplification to obtain the final library. Following the completion of the testing phase, the libraries were then subjected to the Illumina platform for the purpose of conducting high-throughput sequencing.

To improve the accuracy of sequencing results, clean reads were obtained for the purpose of subsequent analysis by the following processes: the removal and filtering of connectors, N-containing reads, and low-quality reads in the raw data. In view of the paucity of genomic data available for *H*. *middendorffii*, the resulting clean reads were spliced using Trinity software (2.4.0) in order to generate transcript sequence data [53], which could thus be utilised as references in subsequent analyses. Finally, we obtained the Unigene sequence by means of Corset (4.6) clustering and remove redundancy [54].

### 4.3. Analysis of Transcriptome Data

The clean reads from each sample were aligned to the reference sequence (Ref) using Bowtie2 software (2.5.4, parameter mismatch 0), and subsequently, the gene expression level (FPKM) of each sample was calculated with the help of RSEM software (1.2.15) [55]. DESeq2 (1.6.3) [56] software was used for DEGs analysed and screened, with |log2 Fold change| > 1 and padj < 0.05 as the criteria for differential gene screening. GO and KEGG enrichment analyses were conducted on the identified DEGs using GOseq (1.10.0) and KOBAS software (2.0.12) to obtain the biological functions and metabolic pathways that were significantly associated with the DEGs. Both GO and KEGG enrichment analyses were considered as significant enrichment with a *p*-value ≤ 0.05. TFs classification of DEGs was performed, according to the PlantTFDB database. The functional genes of the ABA and JA signalling pathways were correlated with AP2/ERF, WRKY, MYB, bHLH, NAC, and bZIP for correlation network prediction using the Metware online website [57]. The predicted correlation network plots were then visualised using Cytoscape software (3.9.0).

### 4.4. Validation of Gene qRT-PCR

To verify the accuracy of the data pertaining to the transcriptome, 16 DEGs were subjected to random selection for analysis by qRT-PCR. Primer 5.0 was utilised in the design of specific primers for qRT-PCR, with *HfEF-1α* selected as the internal reference gene. The specific primer information is shown in Table S3. The extracted total RNA was reverse transcribed utilising the All-in-one 1st Strand cDNA Synthesis SuperMix reverse transcription kit (Novoprotein, Shanghai, China). The synthesized cDNA was then employed as a template for qRT-PCR using the NovoStart SYBR qPCR SuperMix Plus kit (Novoprotein, Shanghai, China). The experiment comprised 3 biological replicates and 2 technical replicates for each gene, and the relative expression levels of the genes were calculated by means of the 2^−ΔΔCt^ method [58].

## 5. Conclusions

The present study sought to elucidate the molecular regulatory mechanisms of *H*. *middendorffii* under drought stress. We performed the first transcriptome sequencing analysis on the leaves and roots of *H*. *middendorffii* using 20% PEG-6000 to simulate drought stress. The findings revealed that 8796 DEGs were identified in leaves, while 3401 DEGs were identified in roots. GO enrichment analysis indicated that the DEGs were primarily involved in a variety of biological processes, including signal transduction and energy metabolism. KEGG pathway analyses revealed significant enrichment in plant hormone signal transduction and photosynthesis-antenna proteins pathways in the leaves, while the roots showed significant enrichment in ribosome, cutin, suberine, and wax biosynthesis pathways. A high number of DEGs were enriched in the Ap2/ERF, WRKY, MYB, bHLH, NAC, and bZIP TF families. Correlation network prediction analysis revealed that genes such as *PP2C*, *JAZ*, and *SnRK2* may be regulated by TFs such as Ap2/ERF, WRKY, MYB, bHLH, NAC, and bZIP to improve drought tolerance in plants. The findings presented here should contribute to a more comprehensive insight into the regulatory mechanisms of *H*. *middendorffii* under drought stress. Furthermore, these findings should provide genetic resources for the further excavation of candidate drought-resistant genes, and, at the same time, promote the development of water-saving garden construction.

## Data Availability

The raw data supporting the conclusions of this article will be made available by the authors on request.

## Supplementary Materials

The following supporting information can be downloaded at: https://www.mdpi.com/article/10.3390/plants14071082/s1, Table S1. Statistics on transcriptome sequencing data of *Hemerocallis middendorffii*; Table S2. Statistics of sequence assembly results; Table S3. The qRT-PCR primer sequence list.
